# Is the current pertussis incidence only the results of testing? A spatial and space-time analysis of pertussis surveillance data using cluster detection methods and geographically weighted regression modelling

**DOI:** 10.1371/journal.pone.0172383

**Published:** 2017-03-09

**Authors:** Boris Kauhl, Jeanne Heil, Christian J. P. A. Hoebe, Jürgen Schweikart, Thomas Krafft, Nicole H. T. M. Dukers-Muijrers

**Affiliations:** 1 Department of Health, Ethics and Society, School of Public Health and Primary Care (CAPHRI), Faculty of Health, Medicine and Life Sciences. Maastricht University, Maastricht, the Netherlands; 2 Department of Sexual Health, Infectious Diseases and Environmental Health, South Limburg Public Health Service (GGD Zuid Limburg), Geleen, The Netherlands; 3 Department of Medical Microbiology, School of Public Health and Primary Care (CAPHRI), Maastricht University Medical Center (MUMC+), Maastricht, The Netherlands; 4 Beuth University of Applied Sciences, Department III, Civil Engineering and Geoinformatics, Berlin, Germany; Universidad Nacional de la Plata, ARGENTINA

## Abstract

**Background:**

Despite high vaccination coverage, pertussis incidence in the Netherlands is amongst the highest in Europe with a shifting tendency towards adults and elderly. Early detection of outbreaks and preventive actions are necessary to prevent severe complications in infants. Efficient pertussis control requires additional background knowledge about the determinants of testing and possible determinants of the current pertussis incidence. Therefore, the aim of our study is to examine the possibility of locating possible pertussis outbreaks using space-time cluster detection and to examine the determinants of pertussis testing and incidence using geographically weighted regression models.

**Methods:**

We analysed laboratory registry data including all geocoded pertussis tests in the southern area of the Netherlands between 2007 and 2013. Socio-demographic and infrastructure-related population data were matched to the geo-coded laboratory data. The spatial scan statistic was applied to detect spatial and space-time clusters of testing, incidence and test-positivity. Geographically weighted Poisson regression (GWPR) models were then constructed to model the associations between the age-specific rates of testing and incidence and possible population-based determinants.

**Results:**

Space-time clusters for pertussis incidence overlapped with space-time clusters for testing, reflecting a strong relationship between testing and incidence, irrespective of the examined age group. Testing for pertussis itself was overall associated with lower socio-economic status, multi-person-households, proximity to primary school and availability of healthcare. The current incidence in contradiction is mainly determined by testing and is not associated with a lower socioeconomic status.

**Discussion:**

Testing for pertussis follows to an extent the general healthcare seeking behaviour for common respiratory infections, whereas the current pertussis incidence is largely the result of testing. More testing would thus not necessarily improve pertussis control. Detecting outbreaks using space-time cluster detection is feasible but needs to adjust for the strong impact of testing on the detection of pertussis cases.

## Introduction

Pertussis is a highly infectious respiratory disease caused by *Bordetella Pertussis* and is especially severe in unvaccinated and incomplete vaccinated children [1]. Despite the implementation of extensive vaccination schemes, the incidence of pertussis is increasing in many countries with a shifting tendency towards adults and elderly [2–7]. In fully vaccinated children and adults with waning immunity, the symptoms are often mild and indistinguishable from other respiratory diseases [5]. The clinical diagnosis of pertussis is challenging, not only because symptoms are often unspecific, but also because co-infection with respiratory diseases complicates diagnosis [5,8,9]. Additionally, sensitivity and specificity of the applied laboratory tests are influenced by vaccination coverage, frequency of mild cases within the population, exposure to pertussis and age of the patient so that no single laboratory test can be considered as “gold standard” for confirming pertussis cases [10]. The lack of universal standards to confirm pertussis infections thus further facilitates the spread of undiagnosed infections.

This is problematic, as transmission through infected, but undiagnosed members of the same household are held responsible for most transmissions to not or incomplete vaccinated infants [11]. To further reduce transmission, several countries such as France, USA and Australia have incorporated adult booster doses in their respective vaccination schemes [12–15] and the Dutch health council recently recommended the introduction of maternal vaccination to the national vaccination program [16].

In the Netherlands, the pertussis incidence is amongst the highest in Europe and rates have increased since 1996 [17]. The underlying reasons of this increase are not fully conclusive. Several studies attribute the increase of pertussis to a waning immunity in adults [2,17] and new emerging strains of *Bordetella Pertussis* [18,19]. Other studies suggest that an increase of detected pertussis infections occurs mainly because of an increased awareness of the population and general practitioners (GPs) [20–22] and enhanced notification systems [21–23].

According to current general practitioner guidelines in the Netherlands, a clinical pertussis diagnosis is considered in patients having typical symptoms such as severe coughing who had contact with a proven pertussis case. Additional testing for pertussis is only recommended for patients in a household with an unvaccinated or incomplete vaccinated child younger than one year old and in households with a woman, which is more than 34 weeks pregnant [24]. For all other groups, testing is rather induced by the patient than the GP [25].

As pertussis is a notifiable disease in the Netherlands [26] and many other countries, the resulting surveillance data on testing and infections is used to monitor changes over space and time [27–29]. Despite previous findings that pertussis is highly heterogeneously distributed in space as well as in space-time [30,31], a substantial amount of current surveillance activities on pertussis is still restricted to a temporal analysis only [7,28,29,32], masking important regional variations and thus complicating an effective public health response.

Geographic Information Systems (GIS) and cluster detection methods–both, purely spatial as well as in time and space have proven useful to locate possible outbreaks of infectious diseases [33–35], including pertussis [30], resulting in a timely and effective response in affected areas. Such an approach might ultimately help to minimize the spread of pertussis at an early stage when the risk of transmission is highest [36].

Efficient pertussis control however, requires additional background knowledge about the determinants of pertussis testing and the determinants of the current pertussis incidence. However, personal patient information such as occurrence of infections within the same household, household composition, vaccination status and socio-demographic characteristics are mostly unavailable on an individual level due to privacy restrictions of surveillance data [37]. Detailed population information on household composition, socio-economic variables and information on available healthcare and infrastructure is in the Netherlands only available on an aggregated level such as neighbourhoods or municipalities [38,39].

In this context, spatial regression models at the ecological level have been increasingly applied in epidemiological studies of infectious diseases in recent years as regression modelling based on aggregated population data allows an analysis of possible risk factors that are unavailable on an individual level [39–41].

Geographically weighted regression modelling (GWR) is an extension of traditional global spatial regression models and measures how the association between disease risk and socio-demographic population characteristics varies over space. This approach often led to the conclusion that the key populations for certain diseases depend largely on the place of residence, resulting in more cost-effective, targeted public health interventions aimed at those groups who are most at risk in specific locations [39,42,43].

This approach has shown to be effective in revealing associated determinants of several infectious diseases such as Hepatitis C [39], HIV [44] and Japanese encephalitis [42] but has also been useful to examine determinants of treatment seeking behaviour i.e. for Malaria [45] and could thus provide a feasible basis to examine the determinants of pertussis testing and the associated determinants of the current pertussis incidence.

The aim of our article is therefore (i) to examine spatial and space-time clustering of pertussis testing, incidence and test-positivity and (ii) to model the associations between socio-demographic, healthcare and infrastructure related determinants and pertussis testing and incidence using geographically weighted regression models.

## Data and methods

### Ethics statement

The medical ethics committee of the Maastricht University Medical Centre (Maastricht, the Netherlands) approved the study (11-4-136) and waived the need for consent to be collected from participants. Since retrospective data originated from standard care (in which one can opt-out for the use of their data for scientific research) and were analysed anonymously, no further informed consent for data analysis was obtained.

### Laboratory data

Pertussis laboratory data were collected between Jan. 1^st^ 2007 and Dec. 31^st^ 2013 in the province Limburg, the Netherlands, comprising a population of 1,121,820 inhabitants [46]. Testing for pertussis was performed by GPs and hospitals in the area and the test-samples were then sent to the six laboratories in the region, which are capable of analysing the collected samples. The data for this study were therefore retrieved from the registries of these six laboratories in the province and included all pertussis tests requested by health care providers. In total, the data consisted of 15,429 tested persons of which 3,312 (21.5%) persons were tested positive. Positivity was either based on the test result of the PCR (5.5% of tests), culture (2.4% of tests) or serology (IgG, 92.2% of tests); for the latter the international standard cut-off value of IgG≥62.5 IU/ml or IgG≥13 VU/ml was used to be a sensitive and specific indicator of a pertussis infection in the past year [47,48]. This standardisation was made, instead of using the laboratory interpretation, because laboratories used test cut-offs that differed between the laboratories and over time. When multiple serology was applied to test a person and test results were inconsistent, the standardised test result was positive when seroconversion occurred from a negative test to a positive test result. In 8.8% (1,361) of the tested persons, standardisation was not possible because IgG-titres were unavailable for the serological test. We were able to filter the laboratory result for most of these tests, but for a total of 1.0% (151) the test result remained missing.

Besides the results of the laboratory diagnosis, the available information included the four-digits postal code, sex, age and date of testing. In total 14,810 tested persons (96.0%) and 3,150 positive persons (95.1%) had valid postal codes assigned and were therefore included in our analyses.

### Outcomes

As outcomes, we examined three different rates: (i) the proportion of tested persons per inhabitants (testing); (ii) the incidence of pertussis expressed as proportion of positive tested persons per inhabitants (incidence) and (iii) the proportion of positive to tested persons (test-positivity). Due to the different vulnerability between age groups [7], four demographic strata were used to calculate the rates outlined above: (i) children aged 0–14, (ii) adults between 15–64, (iii) persons aged 65 and older and (iv), total population. Although infants display the highest vulnerability to pertussis [1], an analysis of pertussis testing and incidence among 0–5 year olds was not advisable due to the low number of tests and infections in this age group.

### Explanatory variables

We assessed several socio-demographic, infrastructure and healthcare-related variables for their association with the proportion of tested persons and pertussis incidence among the different age strata. The data and map sources were available from Statistics Netherlands [49]. The data were available per neighbourhood and had to be aggregated to the four-digits postal codes to match the pertussis laboratory data. In the Netherlands, a neighbourhood is an administrative area within a municipality with a homogenous socio-economic structure [38,39,49]. Due to privacy restrictions of statistics Netherlands, data for each variable is only reported for a minimum of inhabitants or households. For example, the number of persons in a specific age group is only reported for neighbourhoods with more than 50 persons, while average income is only reported for neighbourhoods with more than 100 persons [38,39,49]. The population-weighted aggregation was therefore only based on the neighbourhoods, for which data were made available. The age stratified population data, which were used for the calculation of the proportion of tested persons and the Pertussis incidence, was only available for 2013 from the Central Bureau for statistics Netherlands. As transmission occurs mainly between members of the same households [11], testing between different demographic strata is expected to follow a comparable pattern. We therefore included testing among the other age groups as independent variables in a regression analysis for testing in a specific age group. To analyse the determinants of the incidence, we included the rate of tested persons within the same age group and the rates of infected persons in the other age groups as independent variables. This should in a later stage confirm through GWR in which areas the effect of testing and potential intra-household transmission is stronger than in other areas.

### Exploratory disease mapping

As our study aimed to highlight geographic heterogeneity among different demographic strata and different epidemiological outcomes on a small spatial scale, the numbers in the numerator for each examined demographic strata and epidemiological outcome can be considered as fairly low. This leads to a large variance of the respective rate simply due to varying population densities of relatively arbitrary administrative boundaries. As pertussis testing and infections are highly depending on local characteristics, neighbouring areas can therefore be expected to display similar rates and abrupt changes are more likely to occur due to the effect of relatively arbitrary administrative boundaries—also referred to as the modifiable area unit Problem (MAUP) [50]. We therefore applied a local empirical Bayesian smoothing approach where the respective rates are smoothed towards a local mean. The neighbours were defined as an area sharing a common edge or boundary [51]. The analysis was carried out in GeoDa 1.2.0 [52]. The resulting rates were then imported in ESRI ArcGIS 10.2.

### Local cluster detection

#### Purely spatial cluster detection

The spatial scan statistic is a local cluster test, which identifies the geographic location and statistical significance of local clusters [39,53,54]. The rationale behind a cluster analysis in our study was to detect significant local clusters of high rates within one demographic stratum and compare the location of clusters within the other demographic strata. In this study, we used two different models in SaTScan: For the proportion of tested persons and the incidence of pertussis, we used a purely spatial Poisson model [39,55]. The input data for this model consisted of the number of tested persons / positive tested persons and the population per demographic stratum as well as the centroid coordinates of each postal code [56]. For the proportion of positive tested persons, we used a purely spatial Bernoulli model [54,57]. For this model, the input data consisted of the number of positive tested persons, the number of negative tested persons per demographic stratum and the centroid coordinates for each postal code [56]. The spatial scan statistic then uses a circular scanning window, which is flexible in size up to a user-specified maximum or the standard setting of including up to 50% of the population inside a cluster. The scanning window gradually moves over the coordinates over the study area, evaluating all possible cluster locations and sizes. The statistical significance is evaluated by computing 999 Monte-Carlo replications [58]. In our study, we set the maximum population at risk to be included in a possible cluster not to exceed 5%. This was done since the default settings in SaTScan are more likely to produce very large clusters and therefore contain locations of low relative risk simply because of the circular scanning window [59]. The value of 5% of the maximum population at risk was based on the experience of a previous study in the area, which met our criterion of including only locations of elevated risk in a cluster [39].

#### Space-time cluster detection

A space-time cluster analysis was employed in this study to evaluate whether testing, incidence and test-positivity occur at the same geographical locations and in the same time periods, providing background information whether space-time clusters of pertussis incidence are correlated in space-time with testing.

The space-time cluster analysis in SaTScan is comparable to a purely spatial model, except that the scanning window may be represented as a cylinder, where the base of the cylinder represents the geographic location and the height of the cylinder represents the time component of the scanning window. The scanning window then moves over all centroid coordinates across the study area and evaluates all possible space-time clusters within the study area and study period [60]. Similar to the purely spatial cluster analysis, we used for both, proportion of tested persons and incidence a space-time Poisson model and for the proportion of positive to tested persons, a space-time Bernoulli model [61]. In this study, we used a scanning window that may contain up to 10% of the background population and up to 12 months of the study period. The calculation of purely spatial and space-time clusters was carried out using SaTScan v9.4.1.

#### Selection of explanatory variables

To select a meaningful set of explanatory variables for the regression analysis, we used a data-mining tool called “exploratory regression” in ESRI ArcGIS 10.2. This tool is comparable to a forward step-wise regression. In each step, one additional variable is added to the regression equation and evaluated based on following criteria in our analysis: (i) The regression coefficients are statistically significant (p<0,05) and (ii) do not display multicollinearity (Variance Inflation Factor < 7.5) [62]. We then chose a set of statistically significant explanatory variables as suggested by the exploratory regression that delivered a plausible explanation of the respective outcome (the proportion of tested persons or the pertussis incidence in the respective age group).

#### Geographically weighted poisson regression

We constructed spatial regression models based on the variables suggested by the exploratory regression, which delivered a plausible explanation of the respective outcome. Global spatial regression models are often applied to determine the strength of the association between the dependent variable and a set of explanatory variables, but the obtained coefficients are averaged over the whole study area [51,63]. Our study area however, consisted of 258 postal codes and the socio-demographic composition and available infrastructure varies at local level. It is therefore unlikely that one single coefficient per explanatory variable would be a good estimator of the strength of the association for the whole study area. We therefore favoured a geographically weighted regression (GWR) approach over a global approach. Geographically weighted regression modelling measures how the relationship between a set of explanatory variables and an epidemiological outcome varies over space, resulting in more detailed understanding of the spatially varying key populations and local characteristics of the study area for different epidemiological outcomes [39,41,43,45,64].

The Poisson distribution among the available GWR models is most suitable for diseases, especially if observed counts of cases are low in certain areas [39,65–67]. The dependent variable was in the analysis for testing specified as the number of tests and in the analysis for incidence as positive cases per postal code. The offset variable was specified as the number of inhabitants per postal code. The centroids of each postal code area were used as input coordinates. The geographically weighted Poisson regression (GWPR) calculates an additional global Poisson model to allow a comparison between a global and a local approach. The GWPR uses a kernel function to fit a regression equation for each postal code area, where the centre of the kernel is the regression point. The kernel function assigns decreasing weights to the observations, depending on the distance (bandwidth) of the respective observation to the centre. The bandwidth of the kernel in GWPR can be either fixed or adaptive and the shape of the kernel can follow a Gaussian or a bi-square distribution. The optimization of the bandwidth in a GWPR model can be based on one of the three available criteria: (i) Akaikes Information Criterion (AIC); (ii) Akaikes corrected Information Criterion (AICc) and (iii) Bayesian Information Criterion (BIC) [63,68]. We thus evaluated all 12 possible combinations of kernel shape, bandwidth type and bandwidth optimization method for the eight different dependent variables. The models without clustered residuals were further considered and out of those, the models with the lowest AICc value and highest adjusted R^2^ were then chosen as the final models. The statistical significance of each coefficient per postal code was calculated using pseudo t-values [63]. The statistic behind GWPR is described in detail elsewhere [63].

We assessed clustering of the residuals of the GWPR using the global Moran`s I test in ESRI ArcGIS 10.2. The computation of GWPR was carried out in the GWR4 software [68]. The coefficients were standardized to allow a direct comparison of the strength of association among the examined explanatory variables. To enhance visualization of the spatially varying coefficients, we used the software`s “prediction at non-sample points” function and calculated the predicted values for a grid of Limburg based on a cell size of 100m x 100m. The obtained values were then interpolated using ordinary kriging in ESRI ArcGIS 10.2.

## Results

### Purely spatial analysis

#### Testing

**Fig 1:**
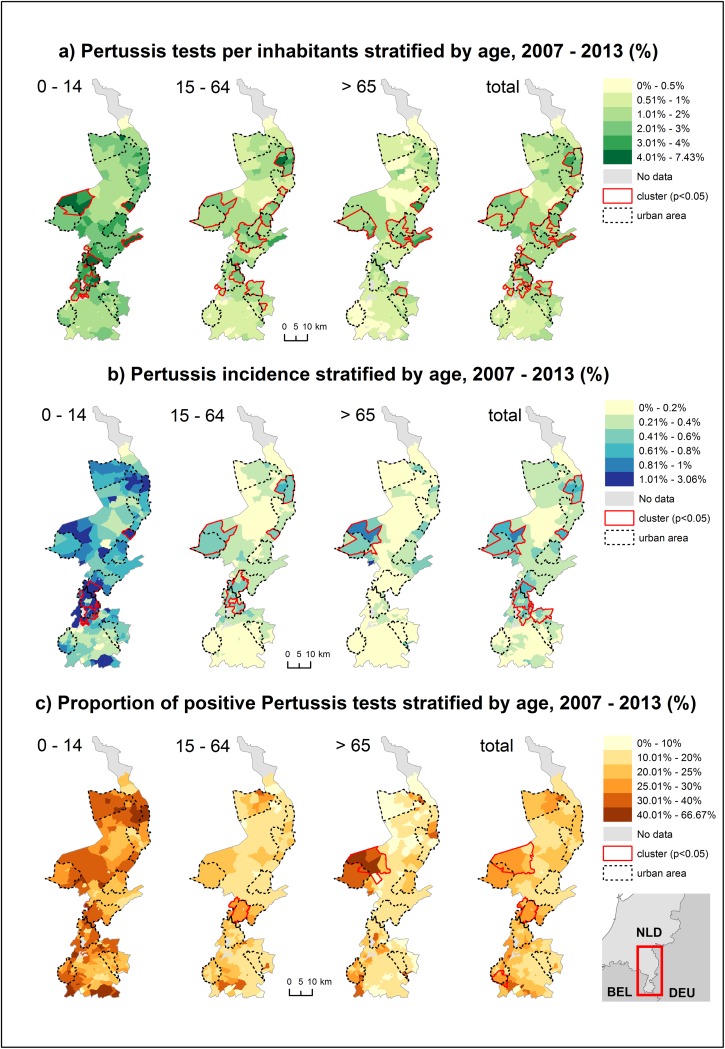
Spatial distribution of a) pertussis testing, b) incidence and c) test-positivity, 2007–2013.

The spatial distribution of the proportion of tested persons among the different demographic strata displayed strong local variations and local clustering (Fig 1). The proportion of tested persons differed widely between the examined age groups and is highest in children (Table 1).

**Table 1:** Rates of testing, test positivity and population incidence. SD = standard deviation.

Age	Tested (%)		Positive tested (%)		Incidence (%)	
	Mean	SD	Mean	SD	Mean	SD
0–14	2.39	1.52	29.77	22.83	0.72	0.70
15–64	1.27	0.75	18.45	13.20	0.23	0.16
>65	0.92	0.93	18.22	17.49	0.16	0.24
All ages	1.36	0.72	21.27	14.09	0.29	0.22

All demographic groups displayed strong local clustering. Despite the differences in the proportion of tested persons, local clustering displayed similar patterns among the different demographic strata. Local clusters were observed especially in the central and northern parts of the study area. In the southern part in contradiction, no clusters could be observed.

#### Incidence

The incidence of pertussis varied between the different demographic groups and was also highest in children (Table 1). Overall, the clusters of the pertussis incidence followed closely the locations of the observed clusters for testing (Fig 1), reflecting a strong spatial correlation to the patterns of testing. The clusters of pertussis incidence among children partially overlapped with clusters among adults and the clusters among adults overlapped in certain areas with those for seniors.

#### Test-positivity

Test-positivity was again highest in children, but the positivity rate did not differ much between adults and seniors (Table 1). Test-positivity in children was the only rate that did not display any local clusters (Fig 1). Among adults, seniors and the total tested persons, only a small number of clusters were observed. Except for one cluster in the southwestern part of the study area for the total tested persons, the clusters observed for test-positivity overlapped with the clusters for testing and incidence.

### Space-time analysis

#### Testing

**Fig 2:**
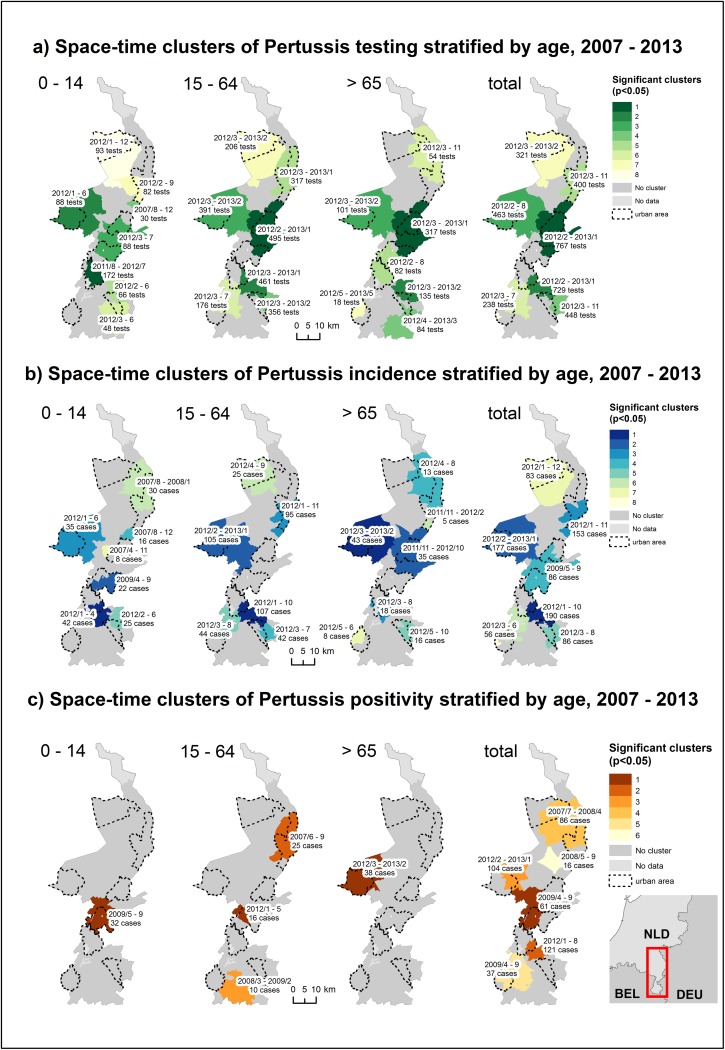
Space-time clusters of a) testing, b) incidence and c) test-positivity, 2007–2013.

In all demographic groups, space-time clustering started generally in the beginning of 2012 and lasted partially until the beginning of 2013 (Fig 2). Only in children, one cluster in 2007 and one cluster starting in 2011 could be observed. Space-time clustering for testing thus started relatively uniform across the study area in the beginning of 2012.

#### Incidence

In adults, seniors and among the total population, the space-time distribution of clusters for the pertussis incidence followed closely the space-time distribution of clusters for testing (Fig 2). The majority of clusters were observed in the beginning of 2012, lasting partially until the beginning of 2013 and were located in the same locations as space-time clusters for testing. In children however, space-time clusters were also observed in 2007 and 2009, for which no space-time clusters for testing were observed.

#### Test-positivity

The distribution of space-time clusters for positivity differed strongly from the observed clusters from testing and pertussis incidence (Fig 2). In children, a cluster observed in 2009 in the centre of the study area overlapped with a cluster for pertussis incidence. In seniors, a cluster observed in 2012 in the northwestern part overlapped with the clusters for testing and pertussis incidence. The other clusters were scattered over the entire study area and study period.

### Geographically weighted poisson regression

#### Determinants of testing

For pertussis testing, the Gaussian kernel type using a fixed, AICc optimized bandwidth fulfilled the requirements of the residuals not displaying spatial autocorrelation among all evaluated demographic groups (Table 2). The local models generally outperformed the global models, reflecting important local differences in the associations between testing and the examined explanatory variables.

**Table 2:** Poisson regression models for pertussis testing, stratified by age. Significance levels: * = 0.05; ** = 0.01; *** = 0.001. Only significant predictors are reported.

	Standardized coefficients of pertussis testing			
Variable	0–14	15–64	>65	Total
**Tested 0–14 (%)**		0.2033***	0.0818**	
**Tested 15–64 (%)**	0.2655***		0.3874***	
**Tested seniors (%)**	0.0659**	0.2360***		
**Children (%)**		0.0610***		0.0525***
**Unmarried (%)**	-0.0418*			
**One person households (%)**		-0.0769***		-0.1567***
**property value (Euro)**	-0.0576***	-0.0419***		-0.1380***
**HH with high income**			-0.0750**	
**Dist. Prim. school (km)**		-0.0354*		-0.0973***
**Dist. GP (km)**		-0.1930***		-0.1367***
**Dist. Pharmacy (km)**		0.0700***		0.0441**
**AICc global**	608	1151	523	2722
**Deviance expl. global**	0.35	0.51	0.40	0.17
**AICc local**	543	697	469	925
**Deviance expl. local**	0.44	0.83	0.49	0.81
**Moran`s I of residuals**	I = 0.06; p>0.05	I = -0,00; p>0,05	I = 0.03; p>0.05	I = -0.01; p>0.05

**Testing in children**: For testing in children, testing in adults was the strongest predictor, followed by a moderate association to testing in seniors. The negative association to unmarried persons reflects that parents, which have ever been married, are a determinant for testing in children. The negative association to mean property value indicates that children in deprived neighbourhoods are more likely to get tested for pertussis.

**Testing in adults:** Testing in children and seniors had the strongest impact on testing in adults. The positive association to household size indicates that adults in multi-person households are more likely to get tested. Similar to testing in children, mean property value was negatively associated. Additionally, testing in adults was associated with proximity to primary schools and GPs, but was also associated with increasing distance to pharmacies.

**Testing in seniors:** Testing in adults had the strongest impact on testing in seniors. The strength of the association to testing in children was relatively weak. Additionally, testing in seniors was negatively associated with proportion of households with high income.

**Fig 3:**
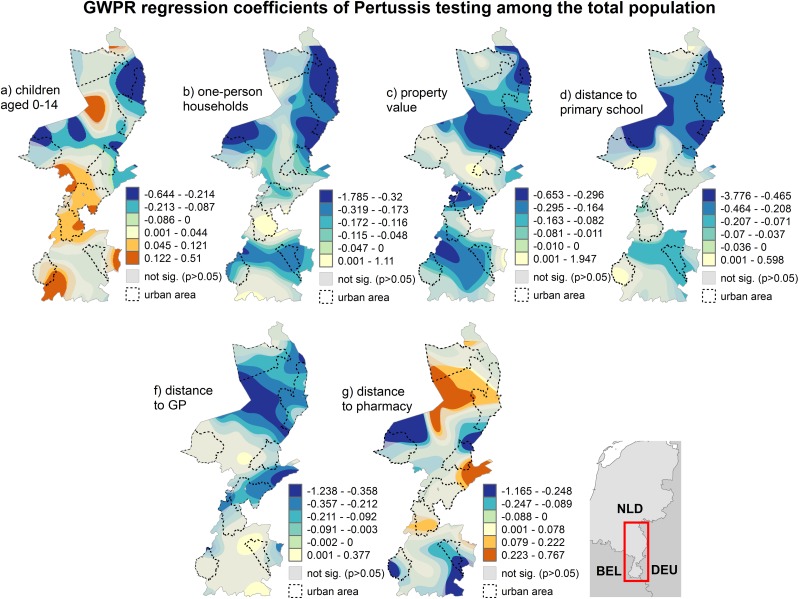
Results of the geographically weighted Poisson regression of pertussis testing among the total population.

**Testing in the total population:** The strongest predictor for testing among the total population was the positive association to household size, followed by the negative associations to mean property value, proximity to GPs and primary schools. The proportion of children and distance to pharmacies were overall positively associated. However, none of the predictors was significant in the entire study area. The results of the GWPR model additionally point out important local variations of the association between testing among the total population and the examined explanatory variables (Fig 3).

#### Determinants of pertussis incidence

For modelling the pertussis incidence, the Gaussian kernel type using a fixed, AIC optimized bandwidth fulfilled the requirements of the residuals not displaying spatial autocorrelation among all evaluated demographic groups (Table 3). The local models outperformed the global models, although the difference was not as pronounced as the difference between global and local models in testing.

**Table 3:** Poisson regression models of pertussis incidence stratified by age. Significance levels: * = 0.05; ** = 0.01; *** = 0.001. Only significant predictors are reported.

	Standardized coefficients of pertussis incidence			
Variable	0–14	15–64	>65	Total
**Tested 0–14 (%)**	0.5191***			
**Tested 15–64 (%)**		0.4425***		
**Tested seniors (%)**			0.5591***	
**Tested total (%)**				0.4692***
**Incidence 0–14 (%)**		0.0819**		
**Incidence 15–64 (%)**			0.3093***	
**Incidence senior (%)**		0.0654**		
**Children (%)**			-	
**One pers. households (%)**		-0.0975***		
**Immigrants (%)**				
**property value (Euro)**				
**HH with low income (%)**				-0.0546**
**Dist. Kinder garden (km)**				
**Dist. Pharmacy (km)**				
**Dist. Hospital (%)**		-0.1481***		-0.0676*
**AIC global**	273	285	275	375
**Deviance expl. global**	0.53	0.60	0.36	0.65
**AIC local**	268	280	249	309
**Deviance expl. local**	0.55	0.65	0.47	0.78
**Moran`s I of residuals**	I = 0.02; p>0.05	I = -0.00; p>0.05	I = -0.04; p>0.05	I = -0.01; p>0.05

**Incidence in children:** In children, testing was the only significant predictor for the pertussis incidence.

**Incidence in adults:** In adults, testing was by far the strongest predictor. The strength of the association to the incidence in children and seniors was comparably small. One-person households were–similar to testing in adults–negatively associated. A significant association to proximity to hospitals could also be observed.

**Incidence in seniors:** Testing was also the strongest predictor in seniors, followed by the incidence in adults.

**Fig 4:**
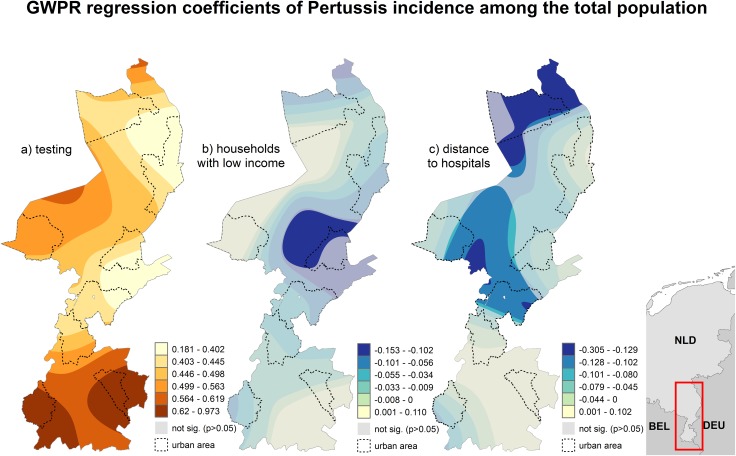
Results of the geographically weighted Poisson regression of pertussis incidence among the total population.

**Incidence in the total population:** Among the incidence for the total population, testing had again the strongest impact on the pertussis incidence. The strength of the negative association to households with low income and proximity to hospitals was comparatively weak. The visualized results of the GWPR model point out that testing was significant in the total study area, despite strong local differences in the strength of this association. The negative association to households with low income and proximity to hospitals was only significant in specific areas (Fig 4).

## Discussion

The main findings of this study are: (i) The determinants of pertussis testing reflect to a certain extent the healthcare seeking behaviour of the general population for common respiratory infections; (ii) the current pertussis incidence is mainly determined by testing and (iii) outbreak detection for pertussis is feasible using surveillance data but needs to adjust for the strong impact of testing.

### Determinants of pertussis testing

Pertussis testing follows to an extent the healthcare seeking behaviour of the general population for common respiratory infections. This can be seen by the associations of proximity to GPs and distance to pharmacies as both compliment each other. Especially in areas where distance to GPs was negatively associated, distance to pharmacies was positively associated. Self-medication for common respiratory infections resulting in coughing is typical in countries where over the counter medication is available [69]. Our results thus could indicate that in areas, where a pharmacy is easily accessible, self-medication for coughing is the first option to choose, whereas in areas where a physician is close, patients with symptoms related to coughing are more likely to consult a GP [70]. As a result, testing for pertussis is to an extent related to treatment-seeking behaviour for common respiratory infections. The association to proximity to primary schools in certain areas indicates potentially an increased awareness of pertussis vulnerability among parents and GPs for children enrolled in primary schools [71]. An overall positive association of pertussis testing among the general population to the proportion of children within the study area was expected as the incidence of pertussis in children is generally higher than in other age groups [72]. The negative association to unmarried persons and mean property value both display a comparable spatial pattern. This could indicate that in families, where the parents have ever been married and are located in a more deprived area are tested more frequently for pertussis, at least in the areas where both associations were significant. Previous studies noted a lower participation rate in childhood immunization schemes among persons with a lower socio-economic status [73]. These associations could therefore be seen as an indicator for an increased awareness of a higher vulnerability to pertussis among this population group, although it has to be noted that the vaccination coverage in the Netherlands is generally very high with approximately 95% [74]. Additionally, higher healthcare use is typical for persons living in deprived areas [75].

When analysing the determinants of pertussis testing in specific age groups, we found that testing in the adjacent age group had the strongest impact on testing in the examined age group. This strong association between the examined age groups corresponds well to previous studies suggesting that intra-household transmission is the most likely route of infection [11,76] and thus logically leads to testing among the same households.

### Determinants of pertussis incidence

The spatial and space-time distribution of clusters for pertussis incidence followed closely the spatial and space-time distribution of pertussis testing, reflecting a strong spatial and space-time correlation between testing and incidence.

We found evidence that space-time clustering of testing for pertussis and the pertussis incidence itself increased across the total study area in the first quarter of 2012, irrespective of the examined demographic group. An increase of diagnosed pertussis cases between 2011 and 2012 has also been noted by the US [77], Great Britain [78] and Spain [79]. When we searched for newspaper articles related to pertussis in the Netherlands within the NexisLexis database (www.nexislexis.com), we found that the number of newspaper articles related to pertussis increased sharply in 2012 as compared to the previous years.

Other studies also suggest a “positive feedback loop” where an increase of diagnosed pertussis cases subsequently causes an increase in testing, which in turn results in an increase of diagnosed pertussis cases again [80,81]. In our study area, the strong increase of testing and pertussis incidence in 2012 could be the result of a combination of several factors; an increased awareness of GPs due to the noted increase in other countries [77–79], stronger media attention and the noted “positive feedback loop” [80,81]. The effect of the “positive feedback loop” is indicated by the fact that space-time clusters for testing and incidence occurred mostly in the same areas and time periods across all evaluated age groups. These findings highlight the importance of monitoring changes in pertussis testing in addition to monitoring the pertussis incidence.

The GWPR analyses confirmed what became already apparent during the spatial and space-time analyses. Regardless of age group, testing was the most important predictor of the pertussis incidence. The second most important predictor for the pertussis incidence in a specific age group was the incidence of pertussis in the adjacent age groups. As our analysis is based on aggregated data, we see these associations as an indicator for intra-household transmission [11,76].

In children, testing was the only predictor for pertussis. No other variables were found to be significant in a regression analysis. Thus, the current incidence among the most vulnerable group does not exhibit any socio-economic associations and can be explained by testing only. In adults, we found an additional association to proximity to hospitals. Increased exposure to pertussis and outbreaks among persons employed in hospitals has been previously noted in several countries such as the US [82] and France [83]. The results of our analysis for adults thus indicate a possible exposure to pertussis in our study area. The positive association to household-size indicates, that for adults living in multi-person households, intra-household transmission might be an important risk factor as well, despite the findings of previous studies that intra-household transmission occurs mainly from parents to children [11,76].

The results of the GWPR model for the pertussis incidence among the total population confirm that testing is the most important predictor for pertussis, despite local differences in the strength of this association. The negative association to households with low income in a small part of the study area was the only significant variable related to socio-economic status. An overall assumption that pertussis is related to specific socio-economic characteristics is therefore not possible. Similar to adults, an association to proximity to hospitals was observed in a small part of the study area. However, further research on an individual level would be necessary to confirm whether an increased pertussis exposure in hospitals exists in this area.

### Outbreak detection using space-time cluster detection

Our results clearly demonstrate that space-time cluster detection is feasible using surveillance data and the methods described in this study. However, in light of the similar increase in testing and incidence in 2012 and its potential association to an increased awareness of the population and GPs, it is necessary to adjust for the impact of testing. This is reflected by the fact that we mostly detected space-time clusters for pertussis incidence in 2012, where the majority of clusters overlap with those for testing. We found only in children clusters in earlier years in 2007–2009, which did not overlap with clusters for testing. It is clear that the incidence of pertussis has to be strongly correlated to testing, no matter which algorithm is used. Logically, pertussis incidence is not suitable in our study area to detect outbreaks. By using test-positivity as indicator however, we could locate space-time clusters within the whole study area and study period, which were not the results of testing only. Test-positivity as indicator is to a certain extent adjusted for testing in a space-time analysis as a similar increase in positive cases and tested persons would not increase the ratio of positive cases to tested persons in the three-dimensional cylindrical scanning window used by the spatial scan statistic. Our results clearly demonstrate that test-positivity could be a better indicator for locating outbreaks, which are not the results of testing only. This conclusion is supported by previous studies, which evaluated the temporal correlation of pertussis incidence to testing behaviour over time [80,81].

When surveillance data–as in our study—are used to locate outbreaks, a prospective space-time cluster analysis using daily updated surveillance data would allow a detection of recent outbreaks in an automated surveillance system as early as possible. Prospective space-time cluster detection has been widely applied in syndromic surveillance [84,85] and an automated, space-time analysis could be implemented for pertussis as well [86].

### Implications for pertussis surveillance

The surveillance data for pertussis in our study were collected by the respective local laboratories and had to merged manually for our study area to allow a retrospective analysis for a whole province. Surveillance of pertussis would thus greatly benefit from an automated near real-time data transfer of each respective laboratory to a centralized institution to allow a detailed, prospective analysis of possible disease outbreaks as early as possible. However, such an approach should focus on a day-wise analysis rather than a month-wise analysis as employed in this study to capture possible outbreaks as precisely as possible [35].

### Strengths and limitations of this study

#### Strengths

First, a major strength of this study is that the design of this study can be repeated for the whole country to confirm whether testing is also in the whole of the Netherlands the most important predictor of pertussis.

Second, the majority of studies on pertussis focus on a purely temporal analysis [7,28,29,32]. Our approach of analysing the spatial and space-time distribution of pertussis therefore provides a novel level of detail as our approach of detecting clusters in space as well as in space-time could allow a more focused detection of outbreaks, resulting in a more timely and cost-effective response.

Third, we analysed the spatial distribution of pertussis testing and incidence at the smallest possible spatial scale, for which surveillance data and population data can be combined without violating privacy restrictions of surveillance data. The four-digit postal code areas are very suitable for small-scale spatial-epidemiological analyses in the Netherlands [38] and thus allow a very detailed analysis of the determinants of pertussis testing and incidence.

Fourth, the use of GWPR allowed us to examine the spatially varying associations between the evaluated outcomes (testing and incidence) and the examined predictor variables. We could therefore clearly see in which areas several predictors such as distance to GPs, pharmacies and hospitals were significant. Our results can therefore be used to allow more targeted investigations—for example—if working in hospitals in the areas outlined in our study comprises an exposure factor on an individual level as well.

Fifth, our study highlighted that the detection of outbreaks using space-time cluster detection is feasible, when using test-positivity as indicator.

#### Limitations

First, we used the international standard IgG cut-off value of 62.5 to consider a diagnosed pertussis infection. As a consequence, the incidence in our study does not necessarily reflect the notification data, which is based on laboratory interpretation.

Second, our analysis was based on aggregated data. The associations detected in our study may not necessarily reflect determinants of pertussis testing and incidence on an individual level. Given the current privacy protection of surveillance data [37], the ecological analysis employed here allowed us to analyse potential determinants that are unavailable on an individual level. Therefore, further research is necessary to confirm whether the associations captured in the ecological analysis really reflect associations on an individual level as well.

Third, we may have missed associations simply because several variables were not available within the data of Statistics Netherlands such as educational level. Lower educational level has been shown to be an important risk factor for poorer health outcomes [87] and could thus also be a possible risk factor for pertussis as well.

Fourth, the GWR4 software currently allows only a purely spatial geographically weighted regression but does not account for space-time differences [63]. Given the strong temporal component of Pertussis testing and infections, a geographically weighted temporal regression might yield more detailed results. However, given the mathematical complexity of such a model [88], this approach could not be implemented in this study.

Fifth, we could not account for the role of vaccinations in our study, as this information is not available in the examined surveillance data. Potentially, data on vaccination among children and booster doses among adults could have a strong impact on the serological test results presented here and could be of additional value for future analyses.

Sixth, we could not verify whether the space-time clusters detected for pertussis incidence or test-positivity capture true outbreaks. However, we think it is reasonable to assume that space-time clusters for the pertussis incidence, which are the results of testing only, are not of interest for the detection of outbreaks. Ultimately, verifying the detected space-time clusters with known outbreaks should be discussed with public health officials residing in the area.

Seventh, we performed a month-wise space-time cluster analysis because a day-wise analysis did consume considerable computation time [35]. Our results are therefore not as temporally precise as it would be technically possible with the surveillance data of our study.

Eight, it would be useful to compare both approaches to detect outbreaks–purely temporal algorithms with the results of the space-time cluster analysis—to evaluate (i) which method allows a more focused outbreak detection and (ii), to evaluate whether the purely temporal algorithms or the space-time cluster detection delivers fewer false alarms. However, such a comparison was beyond the scope of this paper.

## Conclusion

We found empirical evidence that testing for pertussis and the current pertussis incidence in our study area increased similar to other countries in 2012. Testing was higher in deprived areas and was also associated with proximity to primary schools and availability of healthcare. Testing for pertussis thus reflects to an extent the overall healthcare seeking behaviour for common respiratory infections. The pertussis incidence in our study area is largely the result of testing and does not display a specific socio-economic association. The main population at risk for pertussis remains therefore still unknown. Given the strong association of detected pertussis cases to testing, it is questionable whether more testing would enhance pertussis control.

The detection of outbreaks using space-time cluster detection is feasible and could help to facilitate an early and appropriate public health response. However, this approach has to be adjusted for the strong dependency to testing and is probably most efficient when using test-positivity as indicator.
